# Angioleiomyoma of the Sinonasal Tract: A Systematic Review of an Uncommon Clinicopathological Entity

**DOI:** 10.1055/s-0043-1767798

**Published:** 2023-09-26

**Authors:** Gianluca Velletrani, Riccardo Maurizi, Alessandro De Padova, Stefano Di Girolamo

**Affiliations:** 1 Department of Otorhinolaryngology, Università degli Studi di Roma Tor Vergata, Rome, Italy

**Keywords:** angioleiomyoma, sinonasal tract, vascular leiomyoma, angiomyoma, benign nasal tumor, nasal neoplasm

## Abstract

**Introduction** Angioleiomyoma is a rare neoplasm that represents ∼ 0.2 % of all head and neck benign tumors and ∼ 2% of total cases of tumors of the sinonasal tract. It was once considered a possible subtype of leiomyoma, but, in the 2020 World Health Organization (WHO) classification of soft tissue tumors, it is accepted as a singular entity.

**Objective** To systematically review the existing literature on angioleiomyoma in the light of the new classification of soft tissue tumors.

**Data Synthesis** The present study was performed in accordance with the Preferred Reporting Items for Systematic Reviews and Meta-Analyses (PRISMA) statement. A comprehensive search in the PubMed, Cochrane, Scopus, and Google Scholar databases was performed in January 2022. The search items included the following keywords: *nasal angioleiomyoma* OR *sinonasal angioleiomyoma* OR *nasal vascular leiomyoma* OR *sinonasal vascular leiomyoma* . A total of 87 patients were evaluated. He age of the patients in the studies ranged from 15 to 88 years (mean age at diagnosis: 55.6 years). The most common site of involvement was the nasal septum (28.4 %), followed by the inferior turbinate (22.5%). The most common symptom was nasal obstruction (66.7%), followed by epistaxis (47.1%). Surgical excision represented the main treatment, and there was recurrence of pathology in only 1 case (0.9%).

**Conclusion** To our knowledge, only 87 cases of sinonasal-tract angioleiomyoma have been previously described. The results of the present review seem to confirm the rarity and the benign nature of this neoplasm, and they seem to confirm the necessity to improve the available data about sinonasal-tract angioleiomyoma.

## Introduction

Angioleiomyoma (ALM) of the sinonasal tract was originally described in 1966 by Maesaka et al. 1 Until the 2005 WHO Classification of Head and Neck Rumors (third edition), 2 ALM and leiomyoma were considered the same entity, described as a benign tumor of smooth-muscle phenotype. According to this classification, primary leiomyomas of the sinonasal tract seemed to be very rare, with a predilection for the female sex (3.5:1), a peak in the sixth decade of life, and a prevalent location on the turbinates. Other than a positive history of radiant therapy, no risk factors have been reported. The subsequent 2017 WHO Classification of Head and Neck Tumors (fourth edition) 3 mentioned ALM as a possible subtype of leiomyoma with vascular differentiation. In this classification, all leiomyomas showed an equal sex distribution and a prevalence in the adult population; nevertheless, a clear distinction regarding epidemiology, location, and histopathology between the two entities was not reported. The 2020 WHO Classification of Soft Tissue Tumors (5 ^th^ edition) 4 5 for the first time differentiated leiomyoma, of smooth-muscle phenotype, from ALM, of pericytic phenotype.

In the present paper, we have performed a systematic literature review on sinonasal tract ALM to identify previous case reports and to discuss histological features, management, and prognostic aspects of this tumor. Furthermore, we have analyzed possible modifications in the epidemiology, characteristics, and prevalent location of ALM of the data reported in the 2017 WHO Classification of Head and Neck Tumors 3 in comparison to the data reported in the 2020 WHO Classification of Soft Tissue Tumors. 5

## Review of the Literature

The present study was performed in accordance with the Preferred Reporting Items for Systematic Reviews and Meta-analysis (PRISMA)statement. 6

### Search Strategy

A comprehensive search in the PubMed, Scopus, Cochrane, and Google Scholar databases was performed in January 2022, with the collaboration of a medical librarian and without time restriction. The search items included the following keywords: *nasal angioleiomyoma* OR *sinonasal angioleiomyoma* OR *nasal vascular leiomyoma* OR *sinonasal vascular leiomyoma* . The search strategy was created using the Medical Subject Headings (MeSH) intended for PubMed and then tailored to the other databases.

Two independent investigators reviewed the literature found, which was written in English or Spanish. Duplicate articles were removed. Any disagreement regarding inclusion was resolved with a discussion between the two reviewers, and consensus was obtained. After the initial work was completed, the reference lists of the included articles were reviewed to identify and include additional eligible articles. Furthermore, all included studies were meticulously cross-referenced to ensure that patients were not included in multiple articles.

The systematic review was conducted following the PRISMA statement. 6

### Study Selection Criteria

The following were used as inclusion criteria for the present study: studies with subjects of all ages, with a histopathological diagnosis of ALM according to the 2020 WHO Classification of Soft Tissues Tumors, ^5^ written in English or Spanish, and with the full text available. Review articles and commentaries were excluded. The articles were reviewed in full to assess the objectives and level of evidence of the studies. The nature of the present review did not require approval form the Institutional Review Board.

### Data Extraction

The reviewed articles were read in full by two of the authors, and each extracted data using a spreadsheet that included the author(s), the year of publication, the country, the number of patients with ALM, patient characteristics, symptoms, location and size of the tumor, histological subtype, markers, imaging exams, treatment, and follow-up.

### Study Selection

**Fig. 1:**
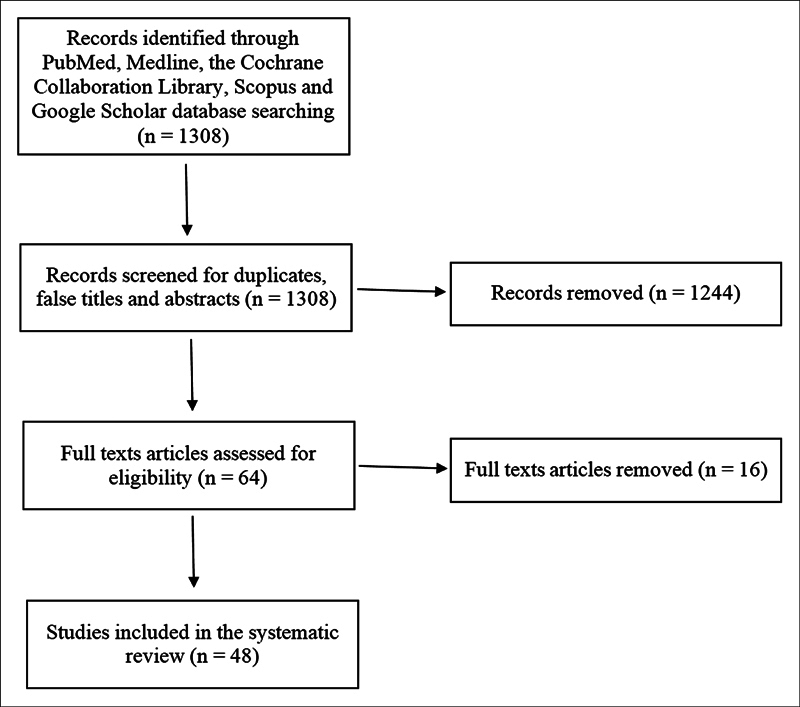
Flowchart showing the systematic review of the literature.

Through the PubMed, Scopus, Cochrane, and Google Scholar databases, 1.308 records were identified ( Fig. 1 ). After the removal of duplicates, false titles, and studies with only the abstract available, 64 records were screened, and 16 were excluded because the full text was not available for 7 records, for another 7 records the language used was not in the inclusion criteria, and 2 records were narrative reviews. We assessed for eligibility 48 full-text articles considering the inclusion/exclusion criteria. The qualitative synthesis included 48 studies, and a case/case series study of sinonasal-tract ALM was reported among them. These studies were published over a period of 48 years, between 1973 and 2021. It is of note that, despite the fact that ALM of the sinonasal tract was originally described in 1966 by Maesaka et al., ^1^ their study was excluded from the present review because the full text was not available.

### Quality Appraisal

To appraise the quality of the included articles, we used the Joanna Briggs Institute (JBI) Critical Appraisal Checklist, 7 which consists of an eight-item scale for case reports and a ten-item scale for case series. The former includes patient demographics, medical history, current clinical condition, description of diagnostic tests, treatment, postintervention clinical condition, adverse events, and the provision of takeaways. The latter evaluates the inclusion criteria, the method of measuring the condition, the validity of the diagnostic methods, whether the inclusion of the participants was consecutive, the completeness of the participants' inclusion, the reporting of the demographics, clinical information, and outcomes, and the appropriateness of the statistical analysis.

### Study Characteristics

Six studies were conducted in the United States, five, in China, four, in India, the United Kingdom, and South Korea, three, in Italy, Japan and Brazil, two, in Germany and Spain, and one study was conducted in each of the following countries: Tunisia, Israel, Switzerland, Taiwan, Greece, Canada, France, Argentina, Saudi Arabia, Colombia, Turkey, and Malaysia. A total of 5 studies were retrospective, and 43 were case reports. The characteristics of the patients are summarized in Table 1 , Table 2 , and Table 3 .

**Table 1:** Clinical characteristics of the cases of sinonasal-tract angioleiomyoma reported in the literature

Reference	Year	Age	Sex	Site	Side	Size (cm)	Symptoms
Schwartzan and Schwartzan 11	1973	57	M	nm	R	nm	Nasal obstruction, headache
Hanna et al. 12	1988	64	F	Inferior turbinate	L	3.0 × 1.2 × 1.0	Nasal obstruction, epistaxis, facial pain,
Sawada 13	1990	41	M	Nasal vestibule	R	nm	No symptoms
Ragbeer and Stone 14	1990	49	F	Anterior nasal floor	R	1.5 × 1.1 × 1.0	Local pain, suppurative rhinorrhea, epistaxis
Khan et al. 15	1994	71	F	Inferior turbinate	L	4.0 × 3.0 × 1.5	Nasal obstruction
Ardekian et al. 16	1996	54	F	Nasal septum	L	1.0 × 2.0	Nasal obstruction, local pain, epistaxis
Nicolai et al. 17	1996	45	F	Ethmoidal sinus	L	7.0 × 3.0	Nasal obstruction
Nall et al. 18	1997	43	F	Superior turbinate	R	nm	Nasal obstrution, epistaxis, facial pain
Murono et al. 19	1998	69	F	Inferior turbinate	R	2.0 × 1.5 × 1.0	Epistaxis
Marioni et al. 20	2002	70	F	Nasal vestibule	R	1.5	Nasal obstruction, epistaxis
Osaki et al. 21	2002	67	M	Nasal septum	L	0.8	Nasal obstruction, epistaxis
Wang et al. 22	2004	70	M	Nasal septum	nm	1.1	Nasal obstruction, epistaxis, pruritus
		66	F	Inferior turbinate	nm	0.3	No symptoms
		62	M	Nasal vestibule	nm	1.5	Nasal obstruction
Burkhardt and Bejarano 23	2006	35	F	nm	R	8.0 × 4,5 × 5.0	Nasal obstruction, epistaxis, nasal dorsum pain, headache, suppurative rhinorrhea, anemia
Chen et al. 24	2007	88	M	Inferior turbinate	R	0.93 × 0.9 × 0.8	Right-side hearing impairment, suppurative rhinorrhea
Campelo et al. 25	2008	44	F	Inferior turbinate head	L	2.2 × 0.9 × 0.7	Nasal obstruction, epistaxis, pruritus
Tas et al. 26	2008	69	M	Ethmoidal and haxillary sinus	R	3.5	Nasal obstruction, tinnitus
Vafiadis et al. 27	2008	68	M	Nasal vestibule floor	R	2.0	Nasal obstruction, right nasolabial line slight bluntness, right upper lip mild swelling
He et al. 28	2009	58	M	Inferior turbinate	R	2.0 × 1.5 × 0.7	Nasal obstruction, epistaxis
Michael and Shah 29	2009	34	M	Inferior turbinate	L	nm	Nasal obstruction, epistaxis
Navarro Júnior et al. 30	2010	62	F	Nasal septum	L	4.0 × 2.0	Nasal obstruction, epistaxis, facial pain
Yoon et al. 31	2013	69	M	Nasal vestibule	nm	1.8	Nasal obstruction
		64	M	Inferior turbinate	nm	0.8	No symptoms
		65	M	nm	nm	1.0	No symptoms
		37	M	Nasal septum	nm	1.0	Nasal obstruction, epistaxis, pruritus
		61	F	Nasal septum	nm	2.0	Nasal obstruction
Arruda et al. 32	2014	49	F	Nasal septum	L	1.6 × 1.5 × 1.1	Nasal obstruction, local pain, nasal scabs, bulging
Yi CH et al 33	2015	70	F	Nasal vestibule	L	1.2 × 0.8	Epistaxis
Burkart and Schoenenberger 34	2015	45	M	Inferior turbinate	L	0.9 × 1.1 × 0.8	Epistaxis, local pain, growing mass
Varghese et al. 35	2015	40	M	Inferior meatus	R	3.0 × 3.0	Epistaxis
Bhandarkar et al. 36	2015	69	F	Middle turbinate	L	4.3 × 2.3 × 3.4	Nasal obstruction, epistaxis, headache, hyposmia
Kim et al. 37	2015	70	F	Inferior turbinate	L	1.6 × 1.2 × 1.4	Nasal obstruction, epistaxis
Agaimy et al. 38	2015	73	M	Nasal cavity lateral wall	R	1.4	Nasal obstruction
		82	M	Inferior turbinate	L	0.8	Epistaxis
		53	M	Nasal vestibule	L	0.8	Growing mass
		76	F	Nasal vestibule	R	0.6	Growing mass
		63	M	Nasal septum	R	0.7	Rhinorrea
		25	F	Ethmoid sinus	nm	0.2	Facial fullness
		77	F	Nm	nm	0.7	Growing mass
		62	F	Nm	nm	1.5	Growing mass
		48	F	Nm	nm	1.2	Growing mass
		26	M	Inferior nasal floor	R	2.5	Local pain
		55	M	Nasal septum	R	1.0	Nasal obstruction, sneezing, difficulty breathing, facial fullness
		77	F	Inferior turbinate	R	0.9	Epistaxis
		51	F	nm	L	1.7	Nasal obstruction, epistaxis, ear pain, cough, growing mass
		36	M	Nasal cavity lateral wall	R	1.7	Growing mass
		65	M	Nasal septum	R	1.0	Nasal obstruction, epistaxis, difficulty breathing
		66	M	Inferior turbinate	R	1.2	Nasal obstruction, epistaxis
Hammedi et al. 39	2015	42	F	Nasal septum	L	1.5 × 1.0	Nasal obstruction, epistaxis
Villarreal Patiño et al. 40	2015	49	M	Middle turbinate	L	nm	Nasal obstruction, epistaxis
Lau et al. 41	2016	43	F	Maxillary sinus	R	nm	Nasal obstruction, epistaxis, facial pain, rhinorrhea
Zhu et al. 42	2016	53	M	Nasal septum	nm	1.0 × 0.5 × 0.3	Epistaxis
		74	F	Nasal cavity lateral wall	nm	2.0 × 1.0 × 1.0	Nasal obstruction
		65	F	Nasal septum	nm	1.5 × 0.5 × 0.5	Nasal obstruction, epistaxis
		55	M	Inferior turbinate	nm	1.0 × 0.8 × 0.5	Nasal obstruction, epistaxis
		62	M	Middle turbinate	nm	1.5 × 1.0 × 1.0	Nasal obstruction, epistaxis
		54	M	Nasal vestibule	nm	1.0 × 0.5 × 0.5	Nasal obstruction
Varadarajan and Justice 43	2016	69	F	Nasal septum	R	1.3 × 1.1	Nasal obstruction, epistaxis
Chen et al. 44	2016	73	M	Nasal septum	L	nm	Nasal obstruction, rhinorrhea
		15	M	Nasal septum	R	nm	Nasal obstruction, epistaxis, local pain
		23	F	Nasal septum	L	nm	Nasal obstruction, headache
		38	F	Nasal septum	L	nm	Nasal obstruction, headache
		45	M	Nasal septum	L	nm	Nasal obstruction, headache
		52	F	Nasal septum	R	nm	Nasal obstruction, epistaxis, local pain
		57	F	Nasal septum	R	nm	Nasal obstruction, epistaxis, local pain
		63	F	Nasal septum	L	nm	Nasal obstruction, headache
		55	M	Nasal septum	R	nm	Nasal obstruction, epistaxis, local pain
		25	M	Nasal septum	L	nm	Nasal obstruction, headache
		67	F	Nasal septum	R	nm	Nasal obstruction, rhinorrhea
		69	F	Nasal septum	L	nm	Nasal obstruction, epistaxis, larynx pain, rhinorrhea
Mathieu et al. 45	2017	54	M	Nasal floor	R	1.0 × 1.0	Nasal obstruction, epistaxis, maxillary sinuses pain
Khanani et al. 46	2017	33	F	Ethmoid sinus	R	3.0 × 3.0 × 1.0	Nasal obstruction, epistaxis
Drapier et al. 47	2019	60	M	Middle turbinate	L	nm	Epistaxis
Lee et al. 48	2019	45	M	Anterior nasal floor	R	1.9	Nasal obstruction, discomfort
Choi 49	2019	30	M	Maxillary sinus	L	3.5 × 3.2	Headache
Apthorp et al. 50	2020	64	F	Mucocutaneous nasal vestibule	R	1.0	Nasal obstruction, alar cartilage swelling
Arora et al. 51	2020	59	M	Between middle and inferior turbinate	L	2.7 × 1.6 × 2.1	No symptoms
Heyman et al. 52	2020	33	M	Inferior turbinate medial border	R	1.3	Facial pain
Ho et al. 53	2020	45	M	Anterior nasal floor	L	2.0 × 1.0 × 1.0	Nasal obstruction, epistaxis
Nada et al. 54	2020	69	M	Anterolateral nasal vestibule	L	nm	Nasal obstruction, jaw and tooth pain, facial fullness
Escamilla Carpintero et al. 55	2021	42	nm	Hasner valve and inside the lacrimonasal duct	L	3.0	Nasal obstruction, epiphora, left eye inner corner bulging
D'Aguanno et al. 56	2021	50	M	Inferior turbinate	L	1.3	Nasal obstruction, facial pain, Left maxillary swelling
		63	M	Inferior turbinate head	L	nm	Nasal obstruction, rhinorrhea
Noreikaite et al. 57	2021	66	M	Nasal septum	R	1.0 × 0.8 × 0.6	Nasal obstruction, difficulty breathing
		52	F	Nasal septum	R	0.4 × 0.4 × 0.2	Epistaxis
Azhdam et al. 58	2021	65	F	Nasolacrimal duct	R	nm	Right eyelid edema, epiphora, styes

Abbreviations: F, female; L, left; M, male; nm, not mentioned; R, right.

**Table 2:** Histological subtype and management of cases of sinonasal-tract angioleiomyoma reported in the literature

Reference	Subtype	Treatment	Follow-up	Imaging
Schwartzan and Schwartzan 11	nm	Excision with transantral ethmoid sphenoidectomy	nm	X-rays
Hanna et al. 12	Solid	Excision of the anterior two-thirds of the inferior turbinate	No recurrence after 1.0 year	X-rays
Sawada 13	Cavernous	Excision	No recurrence after 1.0 year	nm
Ragbeer and Stone 14	Solid	Excision through an incision in the anterior maxillary mucobuccal fold	No recurrence after 1.0 year	X-rays
Khan et al. 15	Venous	Excision with turbinectomy scissors	No recurrence after 1.0 year	CT
Ardekian et al. 16	Venous	Excision	nm	CT
Nicolai et al. 17	Solid	Excision with combined anterior cranial fossa and tranfacial approaches	No recurrence after 2.5 years	CT, MRI
Nall et al. 18	Venous	Embolization and excision with a medial maxillectomy, external ethmoidectomy, and cannulation of the lacrimal system	No recurrence after 1.8 year	CT
Murono et al. 19	Venous	Excision with a margin of normal nasal mucous membrane	nm	CT
Marioni et al. 20	Venous	Excision under local anesthesia with endoscopic control	No recurrence after 0.3 year	nm
Osaki et al. 21	Solid	Excision	No recurrence after 1.3 year	nm
Wang et al. 22	Solid	Excision	nm	nm
	Cavernous	Excision	nm	nm
	Cavernous	Excision	nm	nm
Burkhardt and Bejarano 23	Solid	Embolization and excision with endoscopic surgery	nm	CT
Chen et al. 24	Venous	Excision	No recurrence after 1.0 year	CT
Campelo et al. 25	Venous	Excision with endoscopic surgery	No recurrence after 1.0 year	CT
Tas et al. 26	Solid	Excision with medial maxillectomy and endoscopic ethmoidectomy	No recurrence after 1.3 year	CT
Vafiadis et al. 27	Venous	Excision under local anesthesia through an incision in the gingivolabial sulcus	No recurrence after 2.0 years	X-rays
He et al. 28	Venous	Excision with endoscopic high-power laser cauterization	No recurrence after 1.0 year	CT
Michael and Shah 29	Venous	Excision with endoscopic surgery	nm	CT
Navarro Júnior et al. 30	Venous	Excision with endoscopic surgery	nm	CT
Yoon et al. 31	Venous	Excision	nm	nm
	Solid	Excision	nm	nm
	Solid	Excision	nm	nm
	Cavernous	Excision	nm	CT
	Cavernous	Excision	nm	CT
Arruda et al. 32	Venous	Excision through an incision in the lateral mucosa of the nasal cavity	No recurrence after 2.5 years	CT
Yi CH et al 33	Venous	Excision with endoscopic surgery under local anesthesia	No recurrence after 0.4 years	CT
Burkart and Schoenenberger 34	Venous	Excision with endoscopic surgery with a radiofrequency instrument	No recurrence after 1.0 year	CT, MRI
Varghese et al. 35	Solid	Excision with endoscopic surgery	No recurrence after 0.5 year	CT
Bhandarkar et al. 36	Solid	Excision with endoscopic surgery	Recurrence after 3.0 years	CT
Kim et al. 37	Venous	Excision with endoscopic surgery	No recurrence after 1.0 year	CT
Agaimy et al. 38	Solid	Excision	No recurrence after 4.3 years	nm
	Solid	Excision	No recurrence after 3.6 years	nm
	Venous	Excision	No recurrence after 2.8 years	nm
	Solid	Excision	No recurrence after 2.7 years	nm
	Cavernous	Excision	No recurrence after 2.6 years	nm
	Venous	Excision	No recurrence after 1.3 year	nm
	Solid	Excision	No recurrence after 17.6 years	nm
	Solid	Excision	No recurrence after 6.7 years	nm
	Solid	Excision	No recurrence after 13.4 years	nm
	Solid	Excision	No recurrence after 9.0 years	nm
	Solid	Excision	No recurrence after 4.4 years	nm
	Solid	Excision	No recurrence after 3.8 years	nm
	Cavernous	Excision	No recurrence after 2.2 years	nm
	Solid	Excision	No recurrence after 1.7 year	nm
	Solid	Excision	No recurrence after 1.5 year	nm
	Solid	Excision	No recurrence after 0.8 year	nm
Hammedi et al. 39	Venous	Excision	nm	CT
Villarreal Patiño et al. 40	Venous	Embolization and excision with endoscopic surgery	No recurrence after 3.0 years	CT
Lau et al. 41	Cavernous	Excision with endoscopic surgery	No recurrence after 1.0 year	CT
Zhu et al. 42	Solid	Excision with endoscopic surgery	nm	CT
	Solid	Excision with endoscopic surgery	nm	CT
	Solid	Excision with endoscopic surgery	nm	CT
	Solid	Excision with endoscopic surgery	nm	CT, MRI
	Solid	Excision with endoscopic surgery	nm	CT, MRI
	Solid	Excision with endoscopic surgery	nm	CT
Varadarajan and Justice 43	Venous	Excision with endoscopic surgery	nm	CT
Chen et al. 44	nm	Excision with endoscopic surgery	nm	CT
	nm	Excision with endoscopic surgery	nm	CT
	nm	Excision with endoscopic surgery	nm	CT
	nm	Excision with endoscopic surgery	nm	CT
	nm	Excision with endoscopic surgery	nm	CT
	nm	Excision with endoscopic surgery	nm	CT
	nm	Excision with endoscopic surgery	nm	CT
	nm	Excision with endoscopic surgery	nm	CT
	nm	Excision with endoscopic surgery	nm	CT
	nm	Excision with endoscopic surgery	nm	CT
	nm	Excision with endoscopic surgery	nm	CT
	nm	Excision with endoscopic surgery	nm	CT
Mathieu et al. 45	Solid	Excision with a surrounding rim of normal nasal mucosa through a subgingival incision	No recurrence after 2.0 years	CT, 3D-CT
Khanani et al. 46	Venous	Excision	nm	CT
Drapier et al. 47	Venous	Excision	No recurrence after 1.5 year	CT, MRI
Lee et al. 48	Cavernous	Excision with endoscopic surgery	No recurrence after 0.3 year	CT, MRI
Choi 49	Venous	Excision with Caldwell-Luc surgery combined endoscopic sinus surgery	No recurrence after 2.0 years	CT, MRI
Apthorp et al. 50	Nm	Excision with endoscopic surgery with the two-handed technique	No recurrence after 0.8 year	nm
Arora et al. 51	Solid	Excision with endoscopic surgery with bipolar cautery	nm	CT
Heyman et al. 52	Solid	Excision with endoscopic surgery	No recurrence after 0.5 year	CT
Ho et al. 53	Venous	Excision with endoscopic surgery	No recurrence after 11.0 years	CT
Nada et al. 54	Venous	Excision	nm	CT, MRI
Escamilla Carpintero et al. 55	Venous	Excision with endoscopic surgery with a turbinectomy and exploration of the lacrimal duct	No recurrence after 1.0 year	CT
D'Aguanno et al. 56	Cavernous	Excision with endoscopic surgery	No recurrence after 1.0 year	CT
	Venous	Excision with endoscopic surgery	No reucurrence after 0.4 year	CT
Noreikaite et al. 57	Venous	Excision with endoscopic surgery	No recurrence after 7.0 years	CT
	Venous	Excision with endoscopic surgery	No recurrence after 1.0 year	CT
Azhdam et al. 58	nm	Excision with endoscopic surgery with medial maxillectomy and dacryocystorhinostomy	nm	CT, MRI

Abbreviations: 3D, three-dimmensional; CT, computed tomography; MRI, magnetic resonance imaging; nm, not mentioned.

**Table 3:** Histological markers of the cases of sinonasal-tract angioleiomyoma reported in the literature

Reference	Actin	Desmin	SMA	MSA	SMMHC	Vimentin	Myoglobin	Calponin	S 100	Keratin	AACT	CD31	CD56	CD34	ER	PR	HNF-35	FVIII	HMB45	H-caldesmon	D2–40
Schwartzan and Schwartzan 11	●	●	●	●	●	●	●	●	●	●	●	●	●	●	●	●	●	●	●	●	●
Hanna et al. 12	●	●	+	●	●	●	●	●	●	●	●	●	●	●	●	●	●	●	●	●	●
Sawada 13	●	●	●	●	●	●	●	●	●	●	●	●	●	●	●	●	●	●	●	●	●
Ragbeer and Stone 14	●	+	+	●	●	+	+	●	−	−	−	●	●	●	●	●	●	+	●	●	●
Khan et al. 15	●	●	●	●	●	●	●	●	●	●	●	●	●	●	●	●	●	●	●	●	●
Ardekian et al. 16	●	●	●	●	●	●	●	●	●	●	●	●	●	●	●	●	●	●	●	●	●
Nicolai et al. 17	●	+	+	●	●	+	●	●	●	●	●	●	●	●	●	●	●	●	●	●	●
Nall et al. 18	+	●	●	●	●	●	●	●	●	●	●	●	●	●	●	●	●	●	●	●	●
Murono et al. 19	●	●	+	●	●	+	●	●	●	●	●	●	●	●	●	●	●	●	●	●	●
Marioni et al. 20	+	●	●	●	●	●	●	●	●	●	●	+	●	●	−	+	●	●	●	●	●
Osaki et al. 21	●	+	+	●	●	●	−	●	●	●	●	●	●	+	●	●	+	+	●	●	●
Wang et al. 22	●	●	+	●	●	●	●	●	●	●	●	+	●	●	●	●	●	●	●	●	●
	●	●	+	●	●	●	●	●	●	●	●	+	●	●	●	●	●	●	●	●	●
	●	●	+	●	●	●	●	●	●	●	●	+	●	●	●	●	●	●	●	●	●
Burkhardt and Bejarano 23	+	+	●	●	●	●	●	●	●	●	●	●	●	●	●	●	●	●	●	●	●
Chen et al. 24	●	+	+	●	●	●	●	●	●	●	●	●	●	−	−	−	●	●	●	●	●
Campelo et al. 25	●	●	●	●	●	●	●	●	●	●	●	●	●	●	●	●	●	●	●	●	●
Tas et al. 26	●	●	+	●	●	+	●	●	−	●	●	●	●	+	●	●	●	+	●	●	●
Vafiadis et al. 27	●	●	●	●	●	●	●	●	●	●	●	●	●	●	●	●	●	●	●	●	●
He et al. 28	●	●	●	●	●	●	●	●	●	●	●	●	●	●	−	+	●	●	−	●	●
Michael and Shah 29	●	+	+	●	●	●	●	●	●	●	●	●	●	●	●	●	●	●	●	●	●
Navarro Júnior et al. 30	●	●	●	●	●	●	●	●	●	●	●	●	●	●	●	●	●	●	●	●	●
Yoon et al. 31	+	●	●	●	●	●	●	●	●	●	●	●	●	●	●	●	●	●	●	●	●
	+	●	●	●	●	●	●	●	●	●	●	●	●	●	●	●	●	●	●	●	●
	+	●	●	●	●	●	●	●	●	●	●	●	●	●	●	●	●	●	●	●	●
	+	●	●	●	●	●	●	●	●	●	●	●	●	●	●	●	●	●	●	●	●
	●	●	●	●	●	●	●	●	●	●	●	●	●	●	●	●	●	●	●	●	●
Arruda et al. 32	●	●	●	●	●	●	●	●	●	●	●	●	●	●	●	●	●	●	●	●	●
Yi CH et al 33	●	●	+	●	●	●	●	●	●	●	●	+	●	●	●	●	●	●	−	●	●
Burkart and Schoenenberger 34	●	●	●	●	●	●	●	●	●	●	●	●	●	●	●	●	●	●	●	●	●
Varghese et al. 35	●	−	+	+	●	●	●	●	●	●	●	●	●	●	●	●	●	●	−	●	●
Bhandarkar et al. 36	●	●	+	●	●	●	●	●	+	●	●	●	●	●	●	●	●	●	●	●	●
Kim et al. 37	●	+	+	●	●	+	●	●	●	●	●	●	●	●	−	−	●	●	●	●	●
Agaimy et al. 38	●	+	+	●	●	●	●	●	●	●	●	●	●	●	●	●	●	●	−	+	●
	●	−	+	●	●	●	●	●	●	●	●	●	+	●	●	●	●	●	−	+	●
	●	+	+	●	●	●	●	●	●	●	●	●	●	●	●	●	●	●	−	+	−
	●	+	+	●	●	●	●	●	●	●	●	●	−	●	−	−	●	●	−	+	●
	●	+	+	●	●	●	●	●	●	●	●	●	−	●	●	●	●	●	−	+	−
	●	+	+	●	●	●	●	●	●	●	●	●	●	●	●	●	●	●	−	+	●
	●	−	+	●	●	●	●	●	●	●	●	●	+	●	●	●	●	●	−	+	●
	●	+	+	●	●	●	●	●	●	●	●	●	+	●	●	●	●	●	−	+	●
	●	+	+	●	●	●	●	●	●	●	●	●	+	●	●	●	●	●	−	+	●
	●	+	+	+	●	●	●	●	−	−	●	●	●	●	●	●	●	●	−	●	●
	●	●	●	+	●	●	●	●	−	●	●	●	●	●	●	●	●	●	●	●	●
	●	+	●	●	●	●	●	●	●	●	●	●	●	●	●	●	●	●	−	●	●
	●	+	+	●	●	●	●	●	−	−	●	●	●	−	●	●	●	●	●	●	●
	●	−	+	●	+	●	●	●	−	●	●	●	●	●	●	●	●	●	●	●	●
	●	●	●	+	●	●	●	●	●	●	●	●	●	●	●	●	●	●	●	●	●
	●	+	●	+	●	●	●	●	●	−	●	●	●	●	●	●	●	●	●	●	●
Hammedi et al. 39	●	+	+	●	●	●	●	●	●	●	●	●	●	−	−	−	●	●	●	●	●
Villarreal Patiño et al. 40	●	+	+	●	●	●	●	●	−	●	●	●	●	+	●	●	●	●	●	●	●
Lau et al. 41	●	−	+	●	●	●	●	●	●	●	●	●	●	−	●	●	●	●	●	●	●
Zhu et al. 42	●	●	●	●	●	●	●	●	●	●	●	−	●	−	+	+	●	●	−	●	●
	●	●	●	●	●	●	●	●	●	●	●	−	●	−	+	+	●	●	−	●	●
	●	●	●	●	●	●	●	●	●	●	●	−	●	−	+	+	●	●	−	●	●
	●	●	●	●	●	●	●	●	●	●	●	−	●	−	+	+	●	●	−	●	●
	●	●	●	●	●	●	●	●	●	●	●	−	●	−	+	+	●	●	−	●	●
	●	●	●	●	●	●	●	●	●	●	●	−	●	−	−	−	●	●	−	●	●
Varadarajan and Justice 43	●	●	+	●	●	●	●	●	●	●	●	●	●	●	●	●	●	●	●	●	●
Chen et al. 44	+	●	●	●	●	●	●	●	●	●	●	●	●	●	●	●	●	●	●	●	●
	+	●	●	●	●	●	●	●	●	●	●	●	●	●	●	●	●	●	●	●	●
	+	●	●	●	●	●	●	●	●	●	●	●	●	●	●	●	●	●	●	●	●
	+	●	●	●	●	●	●	●	●	●	●	●	●	●	●	●	●	●	●	●	●
	+	●	●	●	●	●	●	●	●	●	●	●	●	●	●	●	●	●	●	●	●
	+	●	●	●	●	●	●	●	●	●	●	●	●	●	●	●	●	●	●	●	●
	+	●	●	●	●	●	●	●	●	●	●	●	●	●	●	●	●	●	●	●	●
	+	●	●	●	●	●	●	●	●	●	●	●	●	●	●	●	●	●	●	●	●
	+	●	●	●	●	●	●	●	●	●	●	●	●	●	●	●	●	●	●	●	●
	+	●	●	●	●	●	●	●	●	●	●	●	●	●	●	●	●	●	●	●	●
	+	●	●	●	●	●	●	●	●	●	●	●	●	●	●	●	●	●	●	●	●
	+	●	●	●	●	●	●	●	●	●	●	●	●	●	●	●	●	●	●	●	●
Mathieu et al. 45	●	+	+	●	●	●	●	●	−	●	●	+	●	●	●	●	●	●	●	+	●
Khanani et al. 46	●	●	+	●	●	+	●	●	●	●	●	●	●	●	●	●	●	●	●	+	●
Drapier et al. 47	●	●	●	●	●	●	●	●	●	●	●	●	●	●	●	●	●	●	−	+	●
Lee et al. 48	●	+	+	●	●	●	●	●	●	●	●	●	●	●	●	●	●	●	●	●	●
Choi 49	●	●	+	●	●	●	●	●	●	●	●	●	●	●	●	●	●	●	●	●	●
Apthorp et al. 50	●	●	●	●	●	●	●	●	●	●	●	●	●	●	●	●	●	●	●	●	●
Arora et al. 51	●	+	+	●	●	●	●	●	●	●	●	●	●	−	●	●	●	●	●	+	●
Heyman et al. 52	+	+	●	●	●	●	●	+	●	●	●	●	●	●	●	●	●	●	●	●	●
Ho et al. 53	●	+	+	●	●	●	●	●	●	●	●	●	●	●	●	●	●	●	●	●	●
Nada et al. 54	●	●	+	●	●	●	●	●	●	●	●	●	●	●	●	●	●	●	●	●	●
Escamilla Carpintero et al. 55	+	●	+	●	●	●	●	●	●	●	●	+	●	●	●	●	●	●	●	●	●
D'Aguanno et al. 56	●	●	+	+	●	●	●	●	−	●	●	●	●	●	−	−	●	●	−	●	●
	●	●	+	+	●	●	●	●	−	●	●	●	●	●	−	−	●	●	−	●	●
Noreikaite et al. 57	●	●	●	●	●	●	●	●	●	●	●	●	●	●	●	●	●	●	●	●	●
	●	●	+	●	●	●	●	●	●	●	●	●	●	●	●	●	●	●	●	●	●
Azhdam et al. 58	●	●	●	●	●	●	●	●	●	●	●	●	●	●	●	●	●	●	●	●	●

Abbreviations: -, negative; +, positive; ●, not indagated; AACT, alpha-1 antichymotrypsin; ER, estrogen receptor; MSA, muscle-specific actin; PR, progesterone receptor; SMA, smooth-muscle actin; SMMHC, smooth-muscle myosin-heavy chain, D2-40, podoplanin.

A total of 87 patients were evaluated, and their ages ranged from 15 to 88 (mean age at diagnosis: 55.6) years. The male-to-female ratio was of ∼ 1:1 (53.5%, 46 male patients), and data regarding patient sex was not available in 1 case.

The most common site of involvement was the nasal septum ( *n* = 28; 35%), but it also occurred in the inferior turbinate ( *n* = 18; 22.5%), nasal vestibule ( *n* = 11; 13.75%), nasal floor ( *n* = 5; 6.25%), middle turbinate ( *n* = 4; 5%), ethmoidal sinus ( *n* = 3; 3.75%), lateral wall of the nasal cavity ( *n* = 3; 3.75%), maxillary sinus ( *n* = 2; 2.5%), nasolacrimal duct ( *n* = 2; 2.5%), between the middle and inferior turbinates ( *n* = 1), superior turbinate ( *n* = 1), and combined ethmoidal and maxillary sinus ( *n* = 1). Data regarding tumor site was not available in seven cases. The right-to-left side ratio was of ∼ 1:1 (52.2%, 36 cases in the right side), and data regarding the side of the body in which the tumor was located was not available in 18 cases. The dimensional study of the tumor highlighted a mean larger diameter of 1.79 cm (range: 0.2 cm to 8 cm; no data for 21 cases), an average diameter of 1.52 cm (range: 0.5 cm to 4.5 cm; no data for 55 cases), and a smaller diameter of 1.18 cm (range: 0.2 cm 5 cm; no data for 64 cases).

The most common symptom was nasal obstruction ( *n* = 58; 66.67%), followed by epistaxis ( *n* = 41; 47.12%), pain ( *n* = 15; 17.24%; local pain in 9 cases, facial pain in 3 cases, and 1 case of nasal dorsum pain, and 1 case each of jaw and tooth pain and of maxillary sinus pain), headache ( *n* = 9; 10.34%), growing mass ( *n* = 8; 9.19%), rhinorrhea ( *n* = 9; 9.19%; 3 cases with suppurative rhinorrhea), pruritus ( *n* = 3; 3.45%), difficulty breathing ( *n* = 3; 3.45%), facial fullness ( *n* = 3; 3.45%), ear alterations ( *n* = 3; 3.45%; 1 case each of hearing loss, tinnitus, and ear pain), ocular alterations ( *n* = 2; 2.3%; epiphora, styes), and anemia, nasolabial slight, upper-lip swelling, nasal scab, sneezing, hyposmia, larynx pain, alar cartilage swelling, and maxillary swelling ( *n* = 1). In 5 cases (5.75%, 1,15%) no symptoms were reported.

The most common surgical approach was excision ( *n* = 85; 97.7%; in 3 cases, under local anesthesia), followed by excision with a previous embolization ( *n* = 2; 2.3%).

The radiological exams most frequently performed were computed tomography (CT; *n* = 48; 55.17%) followed by both CT and magnetic resonance imaging (MRI + CT; *n* = 9; 10.34%) and X-rays ( *n* = 4; 4.6%). No radiological exams were performed in 26 (29.9%) cases.

The most common histological subtype was solid ( *n* = 32; 44.4%), followed by venous ( *n* = 30; 41.67%), and cavernous ( *n* = 10; 13.89%). Data regarding histological subtype was not available in 15 (17.2%) cases. Histopathologically, the tumor showed immunoreactivity to smooth-muscle actin (SMA; 42/42 cases; 100%), muscle-specific actin (MSA; 7/7 cases; 100%), actin (21/21 cases; 100%), desmin (25/30 cases; 83%), h-caldesmon (13/13 cases; 100%), vimentin (6/6 cases; 100%), FVIII (3/3 cases; 100%), CD56 (4/6 cases; 66.5%), CD31 (7/13 cases; 53%), CD34 (cluster of differentiation) (3/14 cases; 27.3%), myoglobin (1/2 cases; 50%), calponin (1/1 case; 100%), smooth-muscle myosin-heavy chain (SMMHC; 1/1 case; 100%), estrogen receptor (ER; 5/14 cases; 35.7%), progesterone receptor (PR; 7/14 cases; 50 %), S100 (1/11 cases; 9%), no immunoreactivity to HMB45 (a monoclonal antibody that reacts against an antigen present in melanocytic tumors) (0/23 cases), keratin (0/4 cases), D2–40 (podoplanin) (0/2 cases), alpha-l antichymotrypsin (AACT; 0/1 case), and HHF-35 (a muscle actin-specific monoclonal antibody) (0/1 case). In-situ hybridization for the Epstein-Barr virus (EBV) was performed in 4 (4,6%) cases, no cases EBV infection at the single-cell level were detected. The K _i_ -67 proliferation index was described in 25 cases (28.7%), showing a mean value of 2%.

Follow-up was reported in 49 cases (56.3 %). The mean follow-up period was of 2.7 (range: 0.2 to 17.6) years. Only 1 (0.9%) patient experienced local recurrence after 3 years.

## Discussion

Mesenchymal tumors are often a diagnostic challenge for pathologists. Therefore, the standardization and schematization of the classification of these tumors provided by the 2020 WHO Classification of Soft Tissue Tumors ^5^ were necessary.

In the 2005 WHO Classification of Head and Neck Tumors, ^2^ ALM was considered synonymous with leiomyoma, there was a female predilection, and the turbinates were affected more frequently. In the 2017 WHO Classification of Head and Neck Tumors, ^3^ an equal sex distribution for leiomyoma was described, and ALM was reported as a subtype of leiomyoma. The 2020 WHO Classification of Soft Tissue Tumors ^5^ considered ALM as a separate entity from leyomioma.

According to this classification, half of ALMs are painful, the overall male-to-female ratio is of ∼ 0.7:1, the solid type is significantly more common in women, whereas the venous and cavernous types show a male predominance, and the venous type is reported to be more often involved in the head and neck region. Angioleiomyoma is included in the group of pericytic tumors, which share a perivascular growth pattern, a variable contractile phenotype, and represent ∼ 0.2% of all head and neck benign tumors. Angioleiomyoma is a benign entity that manifests mainly in the subcutis or dermis of the extremities (in 89% of the cases).

The incidence of sinonasal ALM is difficult to establish; to the best of our knowledge, only 87 cases of this neoplasm have been described. In the head and neck region, ALM is very rare (8.5% of the cases), in particular in the sinonasal tract, in which the incidence seems to be of ∼ 2% of total tumors. 8 9 Malignancy has not been described. The male-to-female ratio is of ∼ 1:1 (46 male cases and 40 female cases), and the mean age of the patients is of 55.6 (range: 15 to 88) years. According to the results of the present review, we should consider ALM of the sinonasal tract as a rare tumor of elderly patients, with a peak in the sixth decade of life and no gender preference.

Macroscopically, sinonasal ALM manifests as a reddish/pinkish/bluish/brown/gray globular or polypoidal mass, ovoidal or round, soft, elastic, smooth or wrinkled, mainly hypervascular and painless, with a mucous membrane and a slow pattern of growth. According to the findings of the present study, sinonasal ALM is mainly located in the nasal septum, followed by the inferior turbinate and the nasal vestibule; manifestations in other areas of the sinonasal tract are less frequently reported.

The main differential diagnosis is with myopericytoma, glomus tumor, fibromyoma, leiomyosarcoma, angiofibroma, hemangioma, and angiomyolipoma. Morimoto 10 classified the tumors as follows: solid – when the tumor comprises closely-compacted smooth muscle and abundant blood vessels, which are small and slit-like, the smooth muscle bundles surround the vessels and interdigitate with them; venous – when the tumor lacks compacted smooth muscle bundles and the blood vessels have thick muscular walls of varying size; and cavernous – when the tumor consists of numerous dilated vascular channels and smaller quantities of smooth-muscle bundles, which are difficult to distinguish from the muscular walls of the vessel channels. Considering the total of cases, the solid subtype is the most frequent, while the venous subtype seems to be more frequent in women, and the solid subtype, in men.

Angioleiomyomas classically show positive immunohistochemical markers like actin, SMA, MSA, desmin, vimentin, h-caldesmon, calponin, and negative markers like HMB45, keratin, and S100. It is of note that the only case of sinonasal ALM with S100 positivity reported in the literature is also the only case with local recurrence. 36 About the hormonal aspect, the search for ER and PR shows a heterogeneous attitude among the investigated cases. The same thing can be said for CD31, CD34 and CD56.

Surgical excision is the best treatment choice. To the best of our knowledge, only 1 (1.15%) case of sinonasal ALM experienced recurrence during the follow-up after surgical excision. The longest follow-up was of 17.6 years. No malignant transformations or metastases have been reported. According to the results of the present review, prognosis is excellent after the removal of the tumoral mass.

## Final Comments

Angioleiomyoma is a relatively new entry in the classification of soft-tissue tumors classification, and due to its biological and embryological features, it should be considered a distinct tumor entity from leiomyoma. Regarding the sinonasal tract, to the best of our knowledge, only 87 cases of ALM have been described.

The male-to-female ratio of sinonasal ALMs is of ∼ 1:1, with a mild male prevalence. The nasal septum is the most frequently affected site, and pain is present in a small portion of cases. As reported in the previous classification, ^3^ the tumor cells are diffusely and strongly immunoreactive to actin, desmin, h-caldesmon, calponin, and vimentin, and the K _i_ -67 index is usually < 5%.

According to the results of the present study, S100 positivity seems to be associated with tumor recurrence, and the most common histological subtype is solid, considering the total of cases, while there is a prevalence of the venous subtype in women and of the solid subtype in men.

Surgical excision is the best treatment choice without additional medical therapies.

The results of the present review confirm the benignity of this tumor and, despite its low incidence, ALM must be considered in the differential diagnosis of any sinonasal mass, especially in the nasal septum.
